# Epidemiology and Medication Utilization Pattern of Aortic Dissection in Taiwan: A Population-Based Study

**DOI:** 10.1097/MD.0000000000001522

**Published:** 2015-09-11

**Authors:** Ting-Yu Yeh, Chung-Yu Chen, Jiann-Woei Huang, Chaw-Chi Chiu, Wen-Ter Lai, Yaw-Bin Huang

**Affiliations:** From the School of Pharmacy, Master Program in Clinical Pharmacy (TYY, CYC, YBH); Department of Pharmacy, Kaohsiung Medical University Hospital (CYC, YBH); Department of Surgery, Division of Cardiovascular surgery (JWH, CCC); and Department of Internal Medicine, Kaohsiung Medical University Hospital, Kaohsiung, Taiwan, R.O.C (WTL).

## Abstract

Acute aortic dissection (AD) is a catastrophic condition associated with a high rate of mortality. However, current epidemiological information regarding AD remains sparse. The objective of the present study was to investigate the current epidemiological profile and medication utilization patterns associated with aortic dissection in Taiwan.

In this population-based study, we identified cases of AD diagnosed during 2005 to 2012 in the complete Taiwan National Health Insurance (NHI) Research Database. Patients with AD were identified using the International Classification of Disease, Ninth Revision (ICD-9) code 441.0, and surgical interventions were defined using NHI procedure codes.

A total of 9092 individuals with a mean age of 64.4 ± 15.1 years were identified. The cases were divided into 3 groups: Group A included 2340 patients (25.74%) treated surgically for type A AD; Group B included 1144 patients (12.58%) treated surgically for type B AD, and Group C included 5608 patients (61.68%) with any type of AD treated with medical therapy only. The average annual incidence of AD was 5.6 per 100,000 persons, and the average prevalence was 19.9 per 100,000 persons. Hypertension was the most common risk factor, followed by coronary artery disease and chronic obstructive pulmonary disease. Within 1 year of AD diagnosis, 92% of patients were taking antihypertensive medication. Calcium channel blockers were the most frequently prescribed antihypertensive medication for long-term observation in Taiwan.

The annual trends revealed statistically significant increases in the numbers and percentages of prevalence, incidence, and mortality. Changes in patients’ drug utilization in patterns were observed after AD diagnosis. Our study provides a local profile that supports further in-depth analyses in AD-affected populations.

## INTRODUCTION

Acute aortic dissection (AD) is a life-threatening condition associated with high rates of morbidity and mortality. However, epidemiological information concerning this disease remains scarce. Few epidemiologic studies of AD in general populations have been published, and the incidence of AD appears to have increased over time.^1–5^ According to recent reports, the male-to-female ratio among patients with AD ranges from 1.5 to 2:1. The mean age at AD onset is in the mid-60s.^1–5^

The Stanford system is the most widely used one for AD classification. Unlike type B AD, type A AD involves the ascending aorta. Studies have indicated that type A AD is more common than type B AD.^6^ Surgical intervention is the treatment of choice for type A AD because of the poor prognosis if left untreated. In cases of acute type B AD, continuous pain, uncontrolled hypertension, hemodynamic instability, early aortic expansion, malperfusion, and signs of rupture are indications for surgery.^7^

Medical management is essential for all patients with AD and involves long-term control of systemic blood pressure, thus minimizing aortic wall stress and cardiac contractility. However, little information about medication utilization among patients with AD is available. A previous study based on a Taiwanese population analyzed the risk factors for late events among patients with AD.^5^ Additionally, the drug utilization and clinical manifestations associated with AD remain to be clarified. Accordingly, we used a 7-year nationwide population-based database to investigate the current epidemiological profile and medication utilization patterns associated with AD in a general population.

## MATERIALS AND METHODS

### Data Sources and Epidemiological Profile

The National Health Insurance (NHI) is a single-payer compulsory health insurance program that provides universal medical care to all citizens. By June 2014, 23 million people in Taiwan were enrolled in this program, yielding a coverage rate >99.5%. In this population-based retrospective study, all healthcare records logged from January 1, 2004 through December 31, 2012 were extracted from the complete computerized NHI Research Database (NHIRD) in Taiwan. Patients with AD were identified using the International Classification of Disease Ninth Revision code 441.0 (ICD-9). Each patient's medical record included various details, such as demographic data, outpatient, ambulatory, hospital inpatient care, medications, and reimbursement fees. In 2004, the NHI covered surgical procedures for AD. Surgical intervention was defined using NHI procedure codes (69024B, 69035B, 69036B, 69037B).

The NHIRD database did not provide information about the type of AD for cases that did not involve surgical intervention, and therefore, we were unable to define the type of dissection in those cases. In our study, we classified patients with AD using a similar system as that described by Pacini et al.^4^ All patients were divided into 3 groups: Group A included patients with type A AD who underwent surgical procedures involving the ascending aorta, Group B included patients with type B AD who underwent surgical procedures involving thoracic endovascular aortic repair that did not target the ascending aorta, and Group C included patients with any type of AD who received medical therapy alone (no surgical treatment).

The annual AD incidence, prevalence, and mortality rate (per 100,000 individuals) were calculated using the total Taiwanese population each year from 2005 through 2011; these data were published by the Statistical Yearbook of the Interior, Department of Statistics, Ministry of the Interior (http://sowf.moi.gov.tw/stat/year/y02–01.xls). Taiwan can be divided into four geographical regions (northern, central, southern, and eastern). Nearly 45% of the population, the largest proportion, lives in the northern region, followed by the southern (27%), central (25%), and eastern regions (2%). Differences in epidemiological profiles among these geographical regions were analyzed in our study. All patients with AD were followed up through complete healthcare records until death or December 31, 2012 (the end of study period). This study was fully reviewed and approved by an ethical committee.

### Medication Utilization Patterns

The Anatomical Therapeutic Chemical Classification System was used to extract the following data from the NHIRD: drug names, frequencies, dispensing dates, quantities, medication doses, and duration (days) during the study period. We assessed each medication according to defined daily doses (DDDs) of angiotensin-converting enzyme inhibitors (ACEIs), angiotensin receptor blockers (ARBs), β-blockers, calcium-channel blockers (CCBs), α-blockers, vasodilators, diuretics, HMG-CoA reductase inhibitors (statins), aspirin, clopidogrel, warfarin, dipyridamole, and cilostazol. In our study, patients who used a specific medication for over 1 month were defined as taking that medication. Patients exposed to a medication within a short-term period after diagnosis for 30 days were defined as using that medication. The prescribing patterns before and within 1 year after AD diagnosis were investigated and compared in this study.

### Statistical Analysis

The direct standardization method was used to estimate the annual incidence and prevalence of AD. Sex- (per 100,000 population per year) and age-specific rates (5-year age categories) were calculated with respect to the incidence and prevalence. Categorical variables are expressed as frequencies (percentages), and continuous data are expressed as means ± standard deviations. Fisher exact test or the *χ*^2^ test was used to analyze associations between categorical variables, and Student *t* test and an equivalence test were used to compare the means of continuous variables. A Kaplan–Meier analysis was used to plot survival rates, and the differences between the survival curves were assessed using the log-rank test. We also established the significance of annual trends in prevalence, incidence, and mortality using the 2-tailed Cochran–Armitage test for trends. Statistical significance was defined as a 2-sided *P* value <0.05. All statistical analyses were performed using SAS version 9.3 (SAS Institutes, Inc, Cary, NC).

## RESULTS

During the 7-year study period, a total of 9092 patients with AD were identified. The baseline characteristics of these patients are presented in Table 1. Of the patients, 6519 (71.7%) were men, and the mean age at the time of the first episode was 64.4 ± 15.1 years. The most common comorbidity was hypertension (53.87%), followed by coronary artery disease (CAD) (19.71%), chronic obstructive pulmonary disease (COPD) (13.88%), hyperlipidemia (13.1%), and cerebrovascular disease (CVD) (12.46%). Furthermore, 2340 patients (25.91%) in Group A were treated surgically for type A AD, and 1144 patients (12.58%) in Group B were treated surgically for type B AD. An additional 5608 patients (61.68%) in Group C received medical management. The patients who received medical therapy for AD in Group C were older than the patients in other groups who underwent invasive repair procedures for AD. In addition, patients in group C had more severe comorbidities than did patients in the other 2 groups. Patients in group B were found to be significantly more likely to have a history of COPD (11.36%) and CAD (18.62%), compared with the respective rates of 8.59% and 15.47% among patients in group A (*P* < 0.05).

**TABLE 1:**
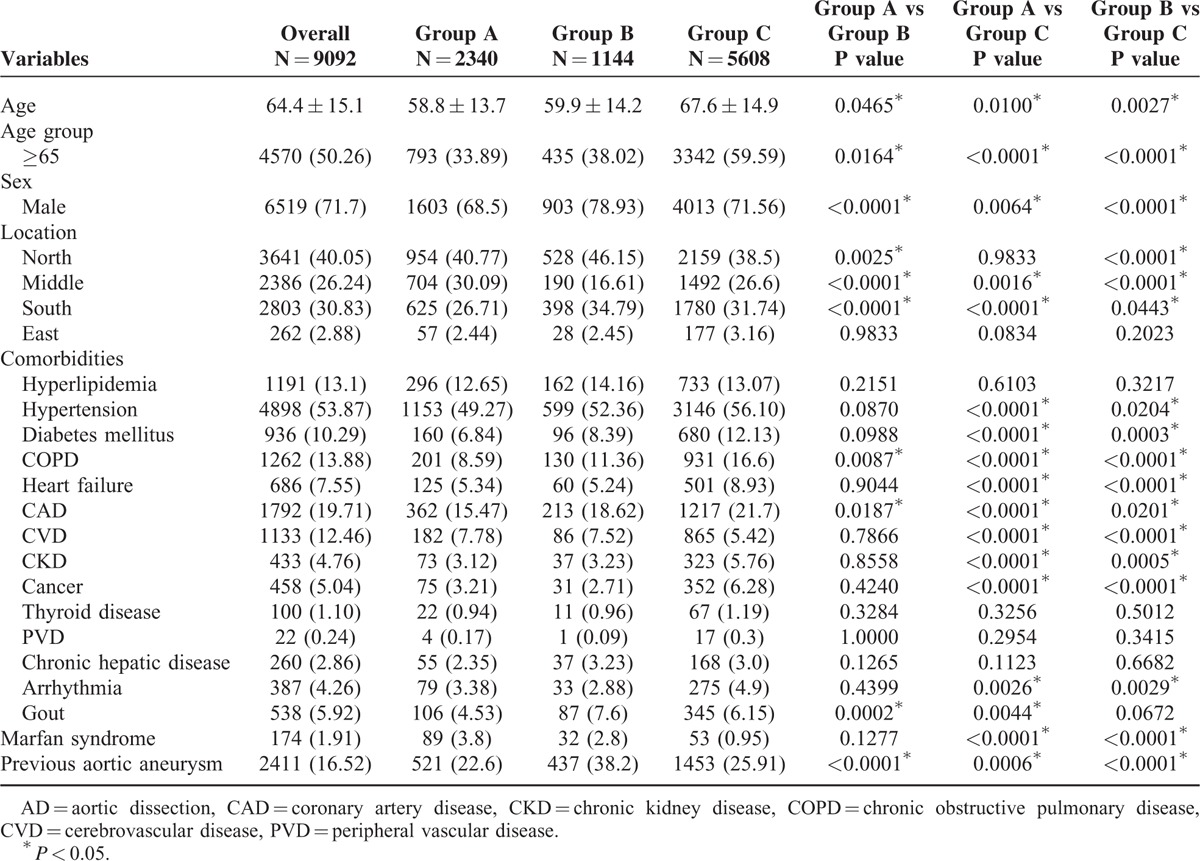
Baseline Characteristics of AD Patients

**FIGURE 1:**
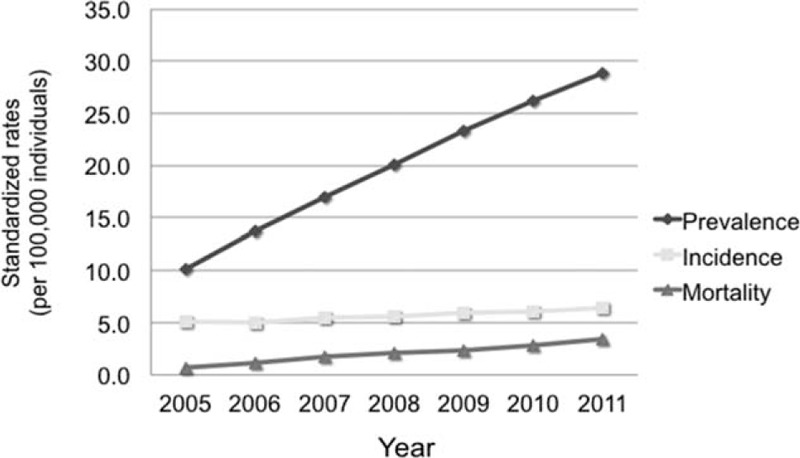
The current epidemiological trend of aortic dissection in Taiwan, among prevalence (*P* value for trend <0.0001), incidence (*P* value for trend <0.0001), and mortality (*P* value for trend <0.0001).

**FIGURE 2:**
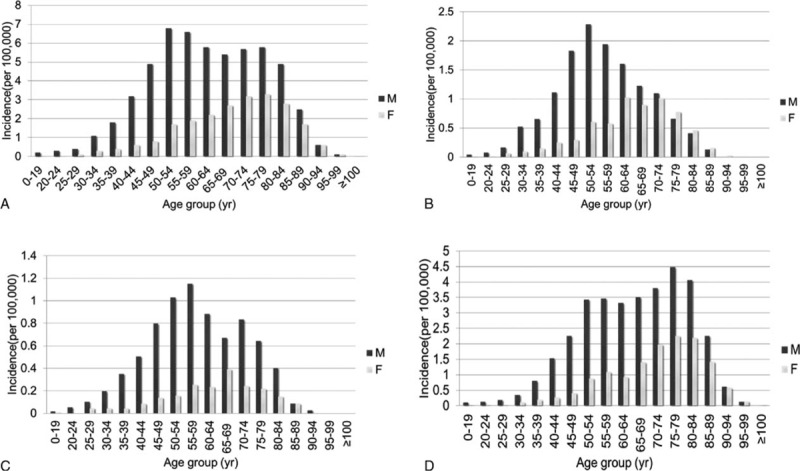
Crude incidence rates (per 100,000 individuals) of all aortic dissections patients in each age group. (A) All population; (B) Group A: surgical intervention type A AD; (C) Group B: surgical intervention type B AD; (D) Group C: receiving only medical treatment without any surgical or interventional procedure. AD = aortic dissection.

From 2005 through 2011, the average annual national incidence rate of AD was 5.6 cases per 100,000 persons. The incidence rate was higher among men than among women (8.0 vs 3.2). Annual incidence trends revealed statistically significant increases in both the number and percentage of cases (*P* < 0.0001, Figure 1). In Group A, the annual incidence rate was 1.4 per 100,000 people, with the peak incidence (2.3/100,000) occurring among males in the 50- to 54-year age group. In Group B, the annual incidence rate was 0.7 per 100,000 people, with the peak rate (1.2/100,000) occurring among males in the 55- to 59-year age group. The incidence among patients in Group C was 3.5 per 100,000 people (Figure 2). During the study period, the annual male:female ratio among AD cases ranged from 2.2 to 3, and the 7-year average ratio was 2.5.

**FIGURE 3:**
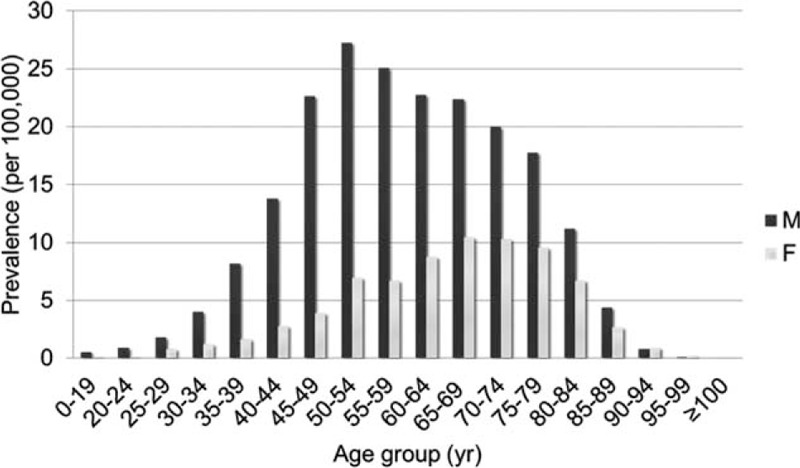
The prevalence of aortic dissection in each age group.

The crude prevalence rates in 2005 and 2011 were 10.1 and 28.9 per 100,000 people, respectively. The average prevalence rate was 19.9 per 100,000 people. In other words, 1 of every 5405 people in the Taiwanese population suffered from AD. The peak prevalence was observed among males in the age range of 50 to 54 years. Over the 7-year study period, the AD prevalence rates exhibited a significantly increasing trend in our population (P < 0.0001; Figure 1). The prevalence rate in group A was 4.9 per 100,000 people, with the peak rate (7.5/100,000) occurring among males in the 50- t0 54-year age group. In Group B, the prevalence rate was 2.9 per 100,000 people, and the peak rate (5/100,000) also occurred among males in the 50- to 54-year age group. The prevalence rate in Group C was 12.2 per 100,000 people, and the peak rate (14.7/100,000) occurred among males in the 55- to 59-year age group. Age-specific prevalence rates among total AD cases are shown in Figure 3.

From 2005 to 2011, the average annual mortality rate associated with AD was 2 per 100,000 people in Taiwan. This rate was higher among men than among women (2.85 vs. 1.2). The annual mortality rate trend exhibited a gradual and significant increase from 2005 to 2011 (*P* < 0.0001; Figure 1). Of the 9092 patients with AD events, 3930 died during the study period. Regarding different geographic regions, the overall AD mortality rate was significantly higher in the north region, compared with the central, south, and east regions (17.44% vs 10.36%, 13.91%, and 1.51%, respectively). Regarding the short-term 30-day and 3-month mortality rates among the surgical intervention patients, group A (15.6% and 17.48%, respectively) had significantly higher rates than group B (12.41% and 14.77%, respectively; *P* < 0.05). However, in terms of long-term 1-year and overall mortality rates, Group A (20.1% and 28.6%, respectively) and Group B (17.5% and 27.8%, respectively) did not differ significantly (Table 2).

**TABLE 2:**
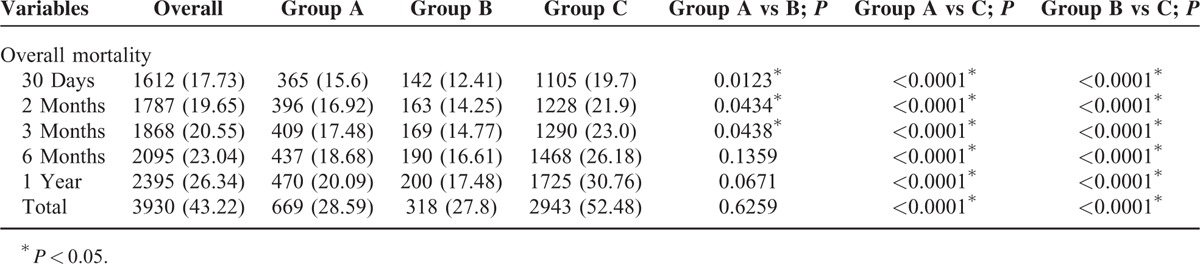
Overall Mortality Within Different Duration of AD Patients

**FIGURE 4:**
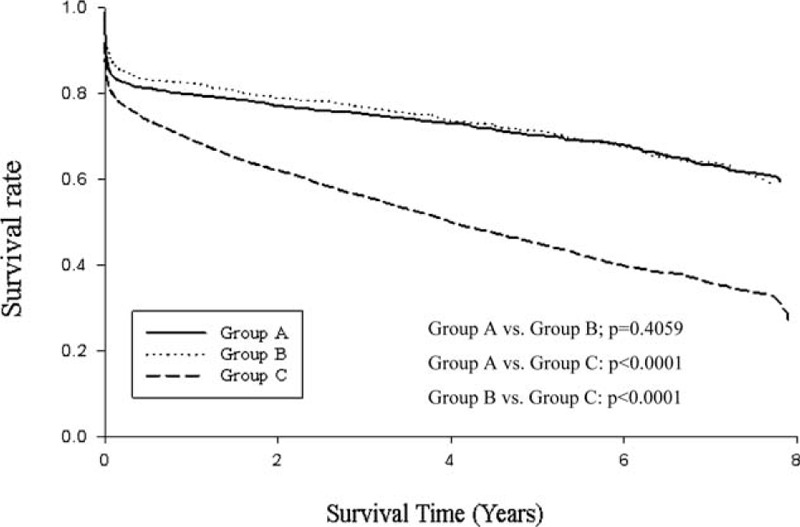
Overall survival rates of the three study groups in 9092 patients with aortic dissection in Taiwan.

During the study period, the overall survival rates at 1, 2, and 3 years were 62.2%, 58.7%, and 55.8%, respectively. In group A, the survival rates at 1, 2, and 3 years were 79.9%, 77.2%, and 75.4%, respectively; the corresponding rates were 82.5%, 79%, and 77%, respectively, for group B and 69.2%, 62.1%, and 56.1%, respectively, for group C. Group C exhibited significant differences in survival when compared with group B or group A (*P* < 0.0001). However, group A and group B did not differ significantly in terms of survival rates (*P* = 0.4059; Figure 4).

Before an AD diagnosis, CCBs were the most frequently prescribed class of antihypertensive drugs (38.21%). An analysis of drug utilization after an AD diagnosis revealed increasing trends in the use of β-blockers, CCBs, ARBs, diuretics, vasodilators, and α-blockers and a decrease in the use of ACEIs (Table 3). Within 30 days of AD diagnosis, however, β-blockers were the most frequently prescribed antihypertensive drug (43.97%), and vasodilators were most commonly used during this short period. The top 3 antihypertensive agents prescribed for long-term observation consistently included CCBs, β-blockers, and ARBs. Before an AD diagnosis, 60% of patients were using some type of antihypertensive medication; within 1 year after an AD diagnosis, approximately 92% of patients were using antihypertensive medication.

**TABLE 3:**
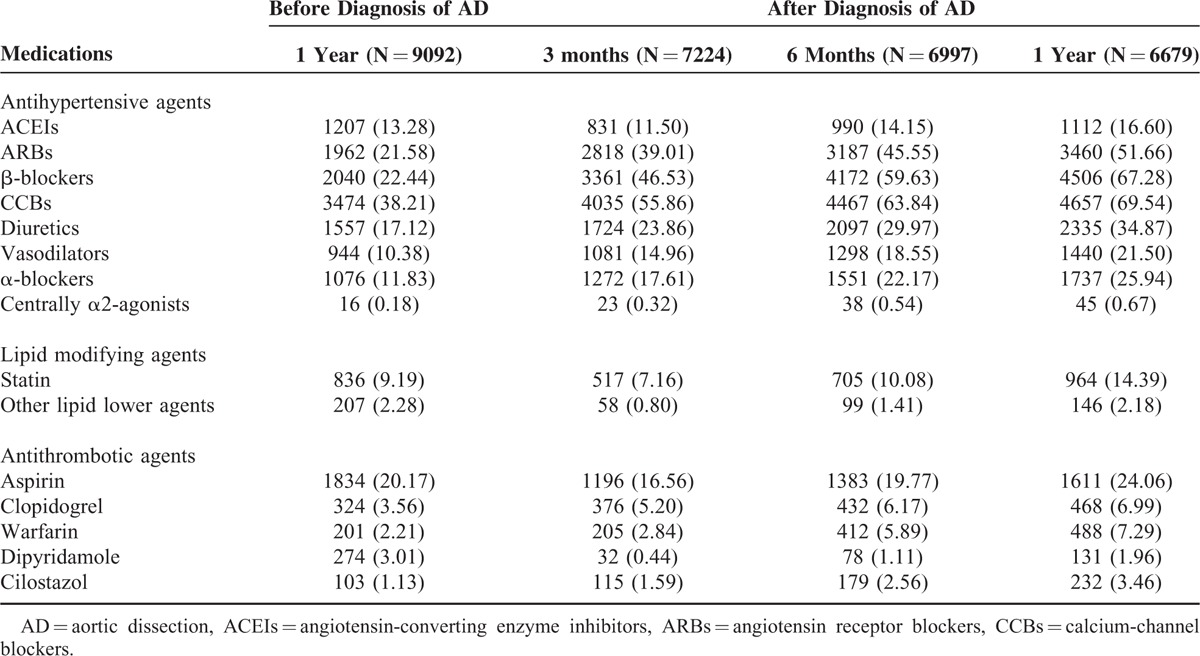
Prescribing Pattern of AD Patients

Statins were the most frequently dispensed lipid modifying agents and were used by approximately 10% of patients with AD. Statin use decreased within 3 to 6 months after diagnosis and increased again after 1 year of observation. However, the use of other lipid modifying agents tended to decrease after an AD diagnosis. Aspirin was the most frequently prescribed antithrombotic agent. Following an AD diagnosis, increases were observed in the use of antithrombotic agents, such as clopidogrel, warfarin, and cilostazol, whereas decreases were observed in the use of aspirin and dipyridamole.

## DISCUSSION

AD is a severe and often lethal emergent condition in people with hypertension. The prognosis of AD is generally poor and depends on the location and expanse of the dissection, the treatment administered, and the time between onset and accurate diagnosis. Patients with type A AD have the worst prognosis, with a 2-fold higher overall in-hospital mortality rate than that of patients presenting with type B AD (30% and 13%, respectively).^8–10^ However, current epidemiological data regarding this catastrophic disease are sparse.

To our knowledge, this is the first population-based data analysis to reveal the current epidemiological profile and medication utilization evaluation among patients with AD. Our study provides an insight into a current cohort of patients with AD and includes longitudinal data from a 7-year study period. Such a large-scale dataset can indicate the annual incidence, prevalence, and risk factors of AD. The annual trends observed during the study period indicated statistically significant increases in the AD incidence, prevalence, and mortality. No other recent epidemiologic studies have reported national prevalence rates. In contrast, our study determined an average prevalence rate of 19.9 per 100,000 persons per year, with a peak prevalence occurring among males aged 50 to 54 years (27.3 per 100,000 persons per year). In Taiwan, most cases of AD occurred in the northern region. Hypertension was the most common risk factor, followed by CAD and COPD. In our country, CCBs were the most frequently prescribed antihypertensive medication for long-term observation.

In our study, AD was more often observed in men than in women (male:female ratio 2.5:1), a ratio larger than those reported in studies from Hungary^3^ (1.5:1), the United States^1^ (1.79:1), UK^2^ (1.48:1), and Italy ^4^(1.8:1). However, the sex distribution was consistent with previous Taiwanese data^5^ (2.17:1). One possible reason for this country-specific difference is that risk factors, such as hypertension, CAD, and smoking, are associated with a high prevalence of AD among Taiwanese men.^11,12^

Previous population-based studies have reported annual AD incidence rates of 3 to 6 per 100,000 persons, and this incidence appears to have increased continuously over time^1–5^ (Table 4). Advanced diagnostic techniques, such as computed tomography and echocardiography, may have contributed to the increased incidence of AD.^13,14^ However, the true incidence of AD is difficult to estimate. Many patients die before reaching a hospital or receiving an accurate diagnosis. We identified an average annual AD incidence rate of 5.6 per 100,000 persons, and this rate increased throughout the study period. Our result is consistent with previous findings.^1,4^ As our dataset included all patients with AD, it is likely to be more inclusive than the datasets used in previous population-based studies. A recent report defined the incidence of variations in arch branching patterns among Chinese patients with AD.^15^ However, few studies have evaluated the frequency of these variations in AD. The influence of this anatomic factor on the pathophysiology of AD requires further investigation.

**TABLE 4:**
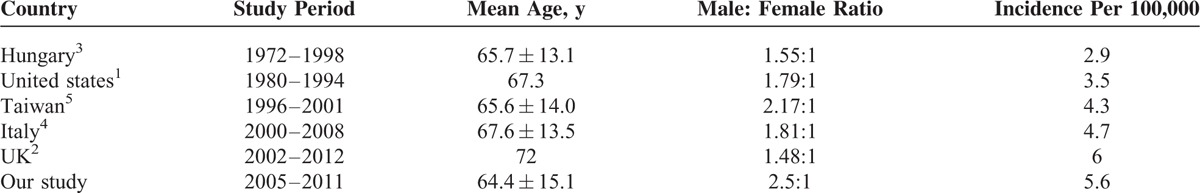
Epidemiologic Studies of Aortic Dissection

We observed a marginally increasing trend in the annual mortality rate from 2005 to 2011. Sidloff et al^16^ reported AD mortality trends indicative of heterogeneity but noted that these rates were generally declining in most western countries. In contrast, increasing rates have been observed in Japan and Romania.^16^ Our study, based on a population of Chinese individuals in Taiwan, yielded results similar to those of a Japanese study.^16^ The differences between countries could be explained by differences in risk factor exposure.^16^ We found that hypertension was the most common risk factor in patients with AD. Recent reports have demonstrated a high prevalence of hypertension in Taiwan, with an approximate rate of 25.6% (29.6% in men and 21.8% in women) during 2013 to 2014.^17,18^ The prevalence of hypertension increased annually and exhibited an increasing trend along with aging.^17,18^ Additionally, the increased awareness of AD and prolonged lifespan of the Taiwanese population may also explain this increasing trend in annual AD mortality.

Some interesting group-specific findings related to mortality emerged; for example, the 30-day and 3-month mortality rates among surgical intervention patients were significantly higher in group A than in group B (*P* < 0.05). However, in long term, 1-year and overall mortality rates did not differ significantly between the groups. A previous report of findings from the International Registry of Acute Aortic Dissection (IRAD) 1996 to 2003 study revealed overall mortality rates of 23% and 21% among surgically treated patients with type A and type B AD, respectively.^9,10^ These reports, however, did not provide the short-term mortality rate information, and therefore, their findings could not be compared with ours. Li et al^19^ investigated AD in 19 large hospitals in China from 2008 to 2011. These authors reported overall mortality rates among surgically treated patients with type A and type B AD of 33.8% and 29.5%, respectively. This result indicated that mortality rates did not differ significantly among surgically treated patients with type A AD and type B AD. The present study findings were in accordance with this earlier result of Li et al's research.^19^ From Figure 4, we also found survival rate of group C patients significantly lower than that of group A and B. In fact, group C patients who were receiving medical therapy were older than other AD patients who underwent invasive repair procedure. The comorbidities of patients in Group C were seen more severe than other 2 groups (Table 1). Therefore, it could be inferred that patients’ condition in Group C was more severe and they were not good candidates for surgery treatment from our long-term survey. Considering the risk for surgery intervention, physicians would rather treat these patients in medical therapy.

The groups of patients with AD in our study were similar to those described by Pacini et al.^4^ The major difference between the studies was that Pacini et al observed a significant difference in the survival rates between groups A and B, in contrast to our study. This difference may be explained by the longer follow-up time and inclusion of all Taiwanese patients with AD in our study. Notably, the survival curves of groups A and B in our study did not overlap within a 3-year period (Figure 4).

During the acute phase of AD, current guidelines initially recommend intravenous β-blockers to reduce the heart rate, ventricular contraction velocity, and blood pressure.^20^ A heart rate of <60 beats per minute and systolic blood pressure of 100 to 120 mmHg are the initial treatment goals. For patients with continuously high systolic blood pressure values, vasodilators may be ideal agents for additional blood pressure control.^21^ We noted that β-blockers were prescribed most frequently, and vasodilators were most commonly used during the acute phase of AD. Accordingly, physicians in Taiwan appear to follow the guideline recommendations when treating patients with AD. Furthermore, Tsai et al,^9^ when using the IRAD data to evaluate long-term survival in patients with type A AD, found that β-blockers were the most frequently prescribed agent, in agreement with our results.

For long-term control, current guidelines suggest that blood pressure should be maintained below 140/90 mmHg through lifestyle changes and the use of antihypertensive medication.^7^ β-blockers have been considered a clinical mainstay of medical therapy for AD.^22–25^ However, our study found that CCBs were the most frequently prescribed antihypertensive products, followed by β-blockers and ARBs. The mechanism underlying the selective effect of CCBs remains unclear. Jonker et al^26^ suggested that the use of CCBs following type B AD may reduce the rate of aortic expansion. Studies in animal models of Marfan syndrome have shown that ARBs inhibit transforming growth factor-β signaling and attenuate progressive enlargement of the aortic root.^27,28^ Recent reports have revealed that ARBs slow down the rate of aortic root dilatation in patients with Marfan syndrome.^29,30^ Further investigations regarding medical management selection are warranted.

Aspirin was the most frequently prescribed antithrombotic agent and was used by approximately 20% of patients with AD. Hansson et al^31^ assessed the use of antiplatelet therapy and its association with bleeding events in patients who underwent surgery for acute AD. In that single-center study, 43 of the 133 included patients (32%) were undergoing antiplatelet therapy; of these, 19 (14%) were using aspirin alone and 24 (18%) were using aspirin and clopidogrel.^31^ The usage prevalence was marginally higher than that observed in our study. Further studies should assess the issue of antithrombotic agent safety in patients with AD.

However, our study had some limitations. First, in the absence of a preceding surgical intervention, we could not distinguish whether patients in our study suffered from type A or type B AD. Accordingly, group C contained patients with both type A and type B AD who were treated medically and whose results could not be compared with those of patients of previous studies. Second, as this study was based on the NHI database, we could not further analyze the aortic diameters, sign and symptoms, or important risk factors, such as smoking and obesity. We observed that COPD and CAD were more frequent comorbidities in this population. This finding may act as a surrogate for other risk factors.^32,33^ Finally, we extracted the AD population using a diagnosis code rather than a practical chart review, and thus, we may have overestimated the incidence and prevalence. Nevertheless, we confirmed the computed tomography (CT) codes and outpatient follow-up data to ensure that the patients were truly affected by AD.

## CONCLUSION

In this nationwide population-based study, the average annual incidence and prevalence of AD in Taiwan were 5.6 and 19.9 per 100,000 people, respectively. Increasing trends in the AD incidence, prevalence, and mortality were observed during the study period. Over 90% patients were using antihypertensive medication within 1 year after an AD diagnosis, and CCBs were the most frequently prescribed drug. An analysis of drug utilization after an AD diagnosis revealed significant increases in the use of β-blockers, CCBs, ARBs, diuretics, α-blockers, clopidogrel, warfarin, and cilostazol. Therefore, our study provides a local profile and facilitates further in-depth analyses of a population of patients with AD.
