# Epidemiology and Medication Utilization Pattern of Aortic Dissection in Taiwan: A Population-Based Study: Erratum

**DOI:** 10.1097/01.md.0000481359.77220.0b

**Published:** 2016-03-03

In the article “Epidemiology and Medication Utilization Pattern of Aortic Dissection in Taiwan: A Population-Based Study”,^1^ which appeared in Volume 94, Issue 36 of *Medicine*, the affiliation for Drs. Yeh, Chen, and Yaw-Bin Huang was originally presented incorrectly. The article has since been corrected online.
